# Computational modelling for congenital heart disease: how far are we from clinical translation?

**DOI:** 10.1136/heartjnl-2016-310423

**Published:** 2016-10-25

**Authors:** Giovanni Biglino, Claudio Capelli, Jan Bruse, Giorgia M Bosi, Andrew M Taylor, Silvia Schievano

**Affiliations:** 1 Bristol Heart Institute, School of Clinical Sciences, University of Bristol, Bristol, UK; 2 Cardiorespiratory Unit, Great Ormond Street Hospital for Children, NHS Foundation Trust, London, UK; 3 Institute of Cardiovascular Science, University College London, London, UK

## Abstract

Computational models of congenital heart disease (CHD) have become increasingly sophisticated over the last 20 years. They can provide an insight into complex flow phenomena, allow for testing devices into patient-specific anatomies (pre-CHD or post-CHD repair) and generate predictive data. This has been applied to different CHD scenarios, including patients with single ventricle, tetralogy of Fallot, aortic coarctation and transposition of the great arteries. Patient-specific simulations have been shown to be informative for preprocedural planning in complex cases, allowing for virtual stent deployment. Novel techniques such as statistical shape modelling can further aid in the morphological assessment of CHD, risk stratification of patients and possible identification of new ‘shape biomarkers’. Cardiovascular statistical shape models can provide valuable insights into phenomena such as ventricular growth in tetralogy of Fallot, or morphological aortic arch differences in repaired coarctation. In a constant move towards more realistic simulations, models can also account for multiscale phenomena (eg, thrombus formation) and importantly include measures of uncertainty (ie, CIs around simulation results). While their potential to aid understanding of CHD, surgical/procedural decision-making and personalisation of treatments is undeniable, important elements are still lacking prior to clinical translation of computational models in the field of CHD, that is, large validation studies, cost-effectiveness evaluation and establishing possible improvements in patient outcomes.

## Introduction

Twenty years ago, pioneering research in the field of modelling congenital heart disease (CHD) showed how numerical simulations based on finite elements method (FEM) and computational fluid dynamics (CFD) could provide insight into local haemodynamics in the total cavopulmonary connection of Fontan patients.^1^ This study beautifully demonstrated how computational results (eg, velocity vector plots, particle path plots, hydraulic dissipation power, energy loss quantification) could generate clinically meaningful data, by simulating different offsets of the Fontan baffle. This work hinted at how simulations could eventually be translated clinically, suggesting that different caval anastomoses designs could be evaluated based on numerical results. Following impressive efforts from the bioengineering modelling community, the use of computational simulations keeps being advocated as a potentially powerful aid in decision-making and treatment. A 2009 study commented on ‘translating the art into science’,^2^ referring to CHD patient-specific models from three-dimensional (3D) imaging reconstructions. But how to translate such science into clinical practice?

The uniqueness and complexity of CHD anatomical arrangements (prerepair and postrepair) warrants a patient-specific approach, which can be facilitated by using computational models. The detailed insight into flow and structural phenomena that models can provide can add to our knowledge of CHD. Recent advances in the imaging realm, for example, 4D cardiovascular magnetic resonance (CMR) imaging flow quantification producing exquisite blood flow visualisation,^3^ have slightly shifted the usefulness of modelling in CHD toward their *predictive* capabilities rather than on haemodynamic insight, that now can be gathered directly in patients.

This review will present engineering tools that can have a relevant role in decision-making, surgical planning and overall pathophysiological appreciation of CHD.

## Personalised healthcare

Computer modelling can advance personalised predictive medicine, whereby an individual's unique anatomy and physiology are used to define the model, predicting outcomes of different treatments and helping to identify optimal strategies.^4^

As mentioned, Fontan surgery has been one of the first modelling applications to CHD. Early work investigated the influence of the Fontan connection on caval haemodynamics as a determinant factor for surgical success.^5^ A study explored 14 different Fontan baffle type options for a single patient, investigating possible postoperative evolutions of the outlet boundary conditions and using optimisation algorithms to identify the theoretically optimal treatment.^6^ Another study applied CFD prior to surgery in order to identify a strategy that could guarantee the best flow distribution to the pulmonary arteries, importantly showing good agreement between predictions and clinical follow-up measurements.^7^ Another example presented a methodology of patient-specific virtual surgery applied to the case of a 6-month old infant, simulating two options for stage II palliation (ie, bidirectional Glenn vs hemi-Fontan operation).^8^ Direct comparison of the outcomes allowed the quantification of the significant changes between preoperative and postoperative conditions. CFD simulations can also be useful to evaluate new technologies/procedures in this context, for example, investigating the design of the Y-graft baffle for Fontan completion on six prospective patients by means of flow simulations.^9^ Results contributed to confirm the reliability of patient-specific simulations, whose outcomes were successfully validated against in vivo data.

Quantification of magnitudes such as blood flow velocity, wall shear stresses (WSSs) and pressure can be critical in planning the treatment of other CHDs where a change in geometry can lead to haemodynamic variations. CFD simulations have been applied to the study of aortic coarctation (CoA), revealing how long-term morbidity can be explained by altered biomechanical indices such as time-averaged WSSs and oscillatory shear index.^10^ CFD can be used to accurately predict the non-invasive pressure gradient across the CoA site without the limitations of simplified assumptions (eg, modified Bernoulli equation in echocardiography), showing good agreement with invasive catheter measurements, and further simulating aortic haemodynamics during stress testing.^11^ Patient-specific modelling can also provide valuable insight to plan the management of pulmonary stenosis, for example, in patients with tetralogy of Fallot. In this case, interventional planning is still exclusively based on detailed anatomical and physiological data derived from imaging and catheter-based modalities.^12^ Simulations could optimise procedural planning by predicting differences in treatment outcome based on individual variability. For instance, a case study presented the use of a model of the proximal pulmonary arteries to investigate changes in flow and pressure following removal of the pulmonary stenosis.^13^

Minimally invasive interventional procedures can benefit from patient-specific predictions for preprocedural assessment, when the operator lacks direct access to the implantation site, choosing the type and size of device based mainly on imaging data. Structural FEM simulations can provide information on the feasibility of device implantation, and have supported minimally invasive procedures such as percutaneous pulmonary valve implantation (PPVI) or CoA stenting.

**Figure 1:**
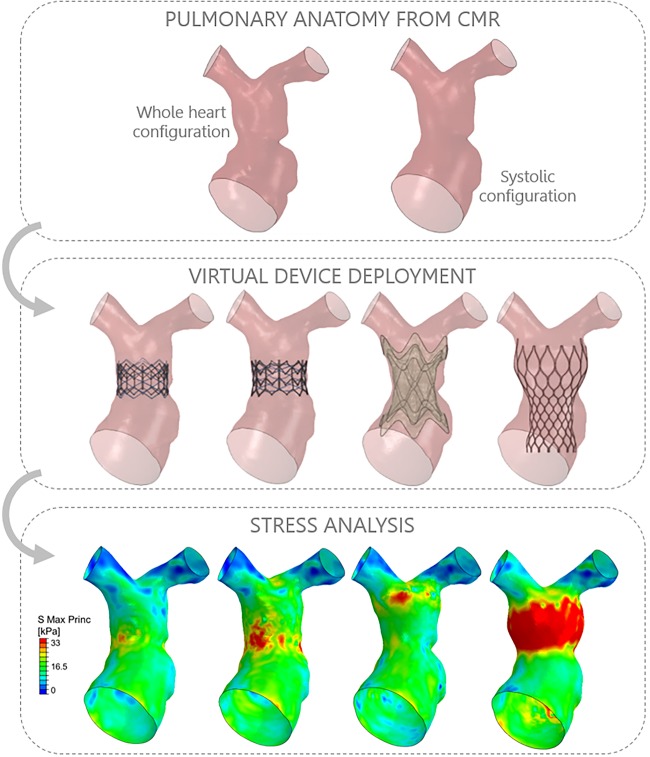
An example of patient-specific simulation for virtual device implantation in the right ventricular outflow tract. The patient-specific anatomy is reconstructed from cardiovascular magnetic resonance (CMR) imaging, taking into account deformations over the cardiac cycle (ie, whole heart configuration in diastole vs systolic configuration). All available devices can be implanted virtually, including simulation of prestenting. Computational analyses can then offer predictions, for example, stresses exerted by the different devices on the vessel wall, aiding in the decision-making process.

**Figure 2:**
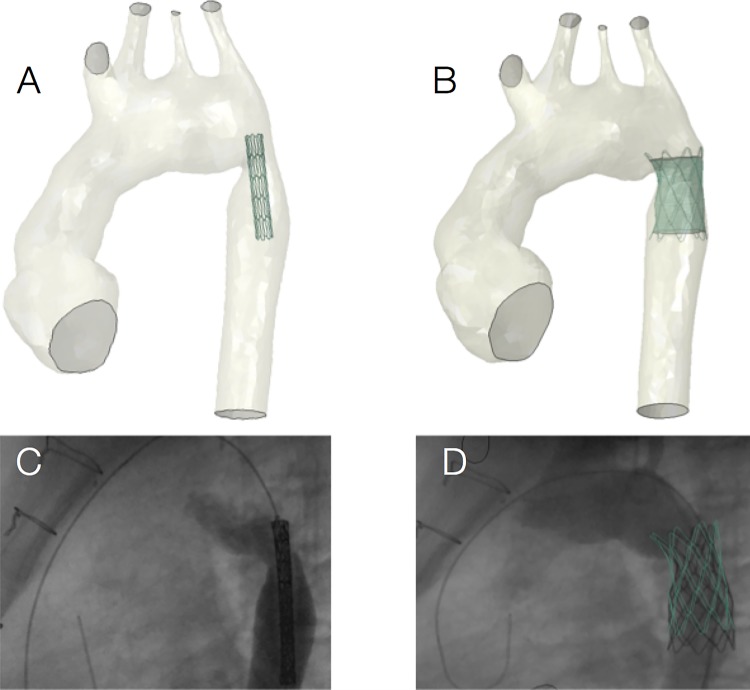
An example of patient-specific simulation for coarctation stenting, showing virtual device positioning (A) and deployment (B), as well as the corresponding fluoroscopy data (C and D) acquired in vivo.

Patient-specific models can be particularly useful for ‘first-in-man’ procedures, for example, implantation of a novel PPVI device.^14^ In this study, simulations were useful to demonstrate the feasibility of implanting the novel self-expanding stent *prior* to the actual procedure. Postprocedural data were then acquired to assess the success of the implantation and were in agreement with computational predictions. This case demonstrates how the patient-specific approach may increase safety of preprocedural planning. Similarly, a modelling paradigm was employed for optimal device selection for a complex PPVI case (figure 1).^15^ A right ventricular outflow tract patient-specific model was used to virtually implant four different percutaneous devices, which theoretically could have all fit the anatomy, assessing their performance (ie, anchoring, migration forces, arterial wall stresses, paravalvular regurgitation). The later comparison with procedural results highlighted the importance of considering the individual implantation site material properties, which may vary considerably across cases. Another example for interventional procedural planning is the case of stenting a complex aortic recoarctation (figure 2), where structural and fluid-dynamic simulations contributed to identify optimal stent size.^16^

So far, these investigations have typically taken the form of case studies or included few patients. A rigorous validation framework based on a large number of cases should be advocated to demonstrate the reliability of the models. Definition of simulations training, certifications and review of the computational techniques are also important,^17^ as standardising CFD and FEM techniques and certifying simulations results, if the latter were used for clinical decision-making and developing new devices or surgical techniques.

## Device development

Computational simulations in the bioengineering industry have been confined to design development of medical devices and instrumentation, somewhat neglecting the interaction between the device and the biological site. However, the potential success or failure of medical devices depends on design characteristics of the device itself, and also on the interaction with the implantation site, requiring knowledge of the dynamic 3D morphology, the anatomic variability between subjects, the interaction forces exerted by the anatomy on the device under physiological/pathological conditions, the long-term mechanical performance of the device when subjected to cyclic in vivo forces, and the biological and mechanical impact of the device itself on the body. In the context of CHD, patient-specific computational modelling can play a fundamental role in the design and testing of new devices as animal models are often inadequate to describe the large anatomical variability encountered in this population,^18^ and bench tests can be very resource-intensive and time-intensive. Compared with adult patients with acquired diseases, children with CHD typically present a wide range of complex anatomies, often repaired in childhood and evolving as the child grows. The development of devices purposely designed for children lags a decade behind device development for adults, as the paediatric market is far smaller, thus less appealing for companies to invest in R&D for such devices. Inexpensive computational modelling appears to be the most promising approach to develop medical devices suitable for patients with CHD.

Patient-specific computational modelling has attracted considerable funding around the world,^19^ and received increasing attention from regulatory agencies due to its potential to significantly reduce the number of manufactured prototypes and animal experiments in phase 0 and 1 trials prior to first-in-man implantation, thus shortening the bench-to-bed pathway. The European Commission sponsored the Virtual Physiological Human initiative since 2006 (http://www.vph-institute.org/, http://www.vph-noe.eu/) and, more recently, the development of a roadmap to introduce *in silico* clinical trials (http://avicenna-isct.org). In 2013, the US Food and Drug Administration (FDA) advocated the use of such systems as an additional innovative research tool,^20^ and created the Medical Device Innovation Consortium with the main purpose of assessing new methods, approaches and standards to enhance the quality and performance of medical devices and improve the timeline of availability of these products to patients. The US Congress recently put forward a bill urging the FDA to engage with device and drug sponsors to explore greater use, where appropriate, of *in silico* trials as these ‘may potentially protect public health, advance personalized treatment, and be executed quickly and for a fraction of the cost of a full scale live trial’.^21^

Third party companies, not directly involved in the Device Industry/Regulatory Agency interaction, but providing services to both by developing computational tools to design cardiovascular devices, are advancing in this direction, for example, leading engineering software company Dassault Systemes Simulia (Providence, Rhode Island, USA). They recently invested in the Living Heart Project initiative to build a computational platform of the human heart including multiphysics capabilities, creating a ‘complete 3D view of electrical impulses and muscle-fiber contractions able to replicate the true motion of the human heart’.^22^ The Living Heart attributes, such as geometry, material properties, loads and boundary conditions, can be modified in order to study cardiac defects and pathologies, and explore treatment options. Devices can be virtually implanted into the model, for example, a novel annuloplasty ring for correction of ischaemic mitral regurgitation,^23^ evaluating effects on cardiac function and predicting mechanical reliability under different conditions. The Living Heart Project just signed a 5-year research agreement with the FDA to test implantation and performance of cardiovascular devices, such as pacemaker leads.^24^

## Statistical shape modelling

Considering CHD as ‘a gross structural abnormality […] of functional significance’ at birth,^25^ the *shape and size* of the heart and its components are crucial for early diagnosis and management of patients with CHD. While non-invasive imaging techniques provide detailed 3D anatomical data, analysis of shape and structure in clinical practice is still often carried out via simple morphometry on 2D image slices, neglecting the abundance of available 3D information. Advances in medical imaging have led to growing image databases and *population-based* studies of cardiac anatomy.^26^ Processing and analysing large amounts of population data combining individual 3D shape information from multiple patients represents a challenge that can be addressed with a statistical shape model (SSM).

**Figure 3:**
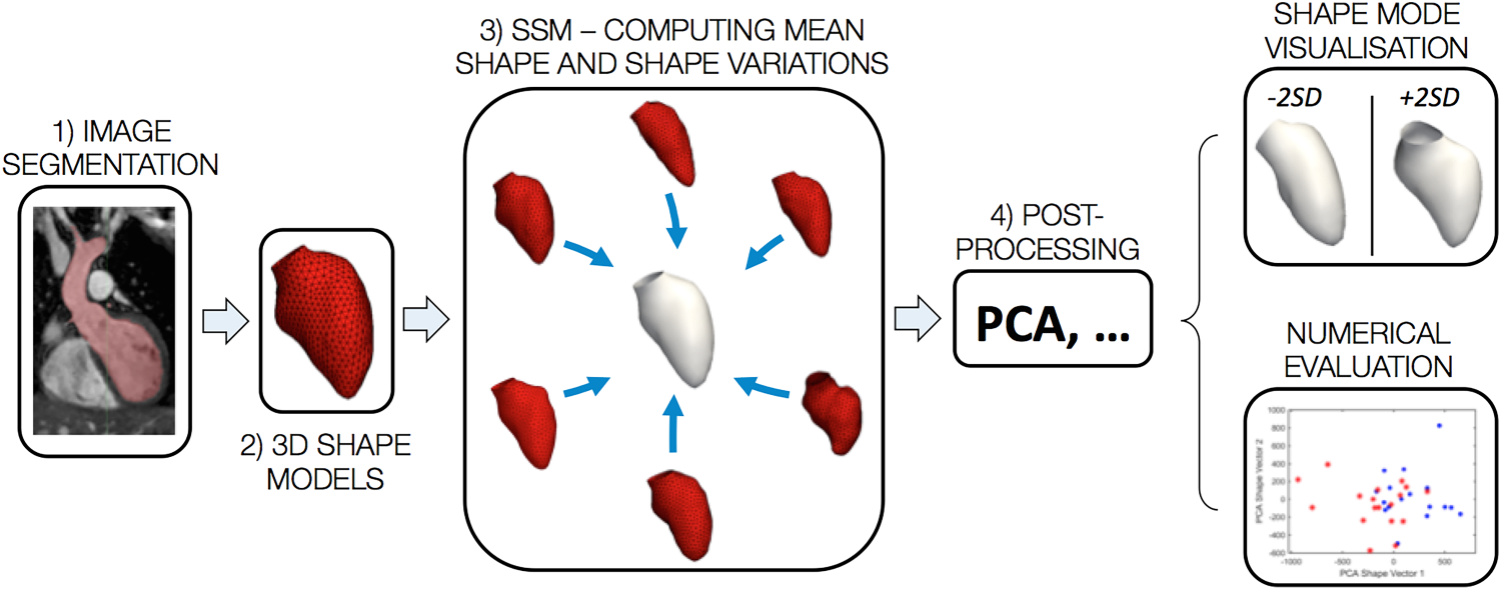
Summarising a statistical shape analysis framework (SSM, statistical shape model). Starting from segmentation of medical imaging data (1), the anatomical segment of interest, for example, the left ventricle, is reconstructed (2) and these two steps are repeated for all the patients in the population, generating the inputs for computing the mean shape (3); postprocessing (4) involves methods such as principle component analysis (PCA), allowing to compare variations in shape (eg, ±2 SD from the mean shape) and perform quantitative assessments.

An SSM is typically based on shapes obtained by segmenting a structure of interest from medical imaging data, providing average anatomical information as a *mean shape* (or ‘template’) and *shape variations* around this mean. The combination of mean shape and its variations constitutes the so-called shape *atlas*, which integrates all shape information for a population of interest.^26^ SSMs can be *descriptive* and *predictive* (figure 3). Descriptive models enable exploration of particular shape characteristics to discover unexpected patterns such as trends, clusters or outliers. Predictive models allow studying relationships between shape and continuous or discrete (clinical or functional) parameters by applying regression or classification techniques.^27^ ^28^

SSMs are already very popular in the field of neuroscience, while cardiac and particularly CHD applications are still limited. Early models described the variability of 2D ventricular shape contours derived manually from echocardiogram images, based on few subjects.^29^ Today, cardiac SSMs range from elaborate 3D models of the whole heart using CT data, to models based on >2000 subjects.^30^ ^31^ Projects such as the *Cardiac Atlas Project* aim to build exhaustive image databases, including CHD scenarios.^31^

SSMs are predominantly used for isolating a structure of interest in medical image segmentation. Yet, not many studies have exploited their capabilities of exploring 3D shape features for diagnostic or prognostic purposes. Examples of studies seeking for ‘shape biomarkers’ include comparison of left ventricular (LV) shape between healthy and diabetic subjects, finding regional LV shape features such as significantly increased septal bulging; analysing ventricular shape in women with pre-eclampsia for risk assessment; and investigating a characteristic LV shape of preterm born subjects.^32–34^ Aortic arch SSMs have explored morphological differences linked to gender and race, and could show associations between sinus shape and valve regurgitation in transcatheter aortic valve replacement (TAVR) patients.^35^ ^36^

In CHD, analysis of right ventricular morphology in patients with tetralogy of Fallot established correlations between distinct ventricular shape features and tricuspid and transpulmonary regurgitation fractions.^37^ Regional LV shape differences have been identified between healthy subjects and patients postarterial switch operation.^38^ Interestingly, SSM results were found to be associated with clinical expert shape assessment of aortic arch morphology in patients with repaired CoA,^39^ suggesting the potential for clinical decision support and diagnosis systems.

Predictive models are very appealing. A computer-aided diagnosis system based on healthy participants and patients with connective aortic tissue disorder used shape modes as classifier for distinguishing between subjects, showing promising classification correctness.^40^ Particularly interesting is a statistical growth model for patients with tetralogy of Fallot able to visualise average right ventricular growth patterns.^37^

The manifold anatomical variety in CHD calls for novel methods to investigate how shape affects function and ultimately patient outcome, so both descriptive and predictive SSMs could be clinically useful in a quest for shape biomarkers. Shape outliers could be automatically detected and followed-up more closely. Clustering techniques could uncover previously unknown shape subgroups, and subsequent classification techniques could explore if any of those subgroups is at a higher risk of following a pathological pathway or growth model. Regression and correlation of distinct shape features with clinical or functional parameters could identify shape biomarkers for adverse cardiac events and even inspire novel surgical approaches for repairing specific morphologies. Growth models could assist in treatment planning. Finally, in relation to above-mentioned *in silico* trials, shape atlases could be used to develop devices that fit the majority or different classes of CHD populations.

As for every statistical model, increasing numbers will strengthen the models, building up on existing small sample studies that often focus on novel algorithms and moving toward larger-scale studies driven by clinical questions. This needs close collaboration between clinical and engineering centres, multicentre studies and pooling data from various imaging modalities or existing models.

## Merging functional and molecular data

Having considered structural, haemodynamic and morphological variables, it is important to remember that a strong interaction exists between mechanical phenomena and biological processes. Computational models can, for example, output measures of WSS, that can be interpreted in the light of thrombus formation (low WSS) or potential vessel rupture (high WSS). A relevant CHD example can be represented by a validated computational model of repaired transposition of the great arteries showing regional increased WSS compared with an age-matched healthy control subject.^41^ Taking this argument further, a recent elegant study looking into patients with bicuspid aortic valve measured differences in WSS between bicuspid and tricuspid scenarios as well as different bicuspid valve fusion patterns, relating regional differences in aortic WSS from in vivo CMR and underlying histological differences.^42^ This is an important observation and leads to considering that, in order to fully exploit the predictive potential of CHD models, it is critical to take into account multiscale processes, especially if considering a time-related phenomenon (eg, effect of flow-related stresses on the aortic wall over time) versus an immediate phenomenon (eg, stent positioning in the implantation site at the instant of deployment). Refined simulations can incorporate such coupling between vascular growth and CFD, by means of a fluid-solid-growth framework.^43^ The application of this type of models to CHD would also require taking into account paediatric blood rheological properties.^44^ Furthermore, a multiscale approach can also be used to model phenomena such as blood clot formation,^45^ which can be relevant in CHD simulations. For instance, in the above-mentioned scenario of Fontan patients receiving a Y-graft, flow simulations can identify possible areas of low WSS leading to potential thrombus formation and obstruction of a limb of the baffle.^9^

While technically challenging and not required for all applications, inclusion of multiscale phenomena can considerably strengthen the power of numerical models, particularly growth models, which in turn could be very relevant for CHD evolution predictions. Some of these associations are known, not just at a histological level (eg, aortic aneurysmal disease and related upregulation/downregulation of several microRNAs).^46^ Integration of such information in the models is particularly interesting for phenomena such as calcification, clotting and changes in arterial wall properties, for example, surgical patches.

## Multimodal data and uncertainty

Recent advances in technologies such as 3D echocardiography can also enrich models, for example, including detailed valve morphologies.^47^ Merging imaging data from different sources (eg, CMR+3D echo) can lead to creating and setting new models with functional and anatomical data from multiple sources.

Another important development is the inclusion of uncertainty analysis and optimisation algorithms in the modelling process. An application of this uncertainty quantification in virtual surgery for patients with single ventricle showed statistical variability in the predictions, importantly allowing including a CI in the simulation results.^48^ Indeed, the fact that simulations would previously output a single result has historically represented a limitation with regards to clinical translation of the models, as clinicians remain sceptical unless models are robustly validated, as realistic as possible, and accounting for naturally occurring uncertainties (including variability of the input data).

Biomechanical modelling is a powerful tool, not just for CHD, and also in other areas of cardiovascular medicine, for example, simulating stent behaviour and stent implantation in coronary arteries in adult patients.^49^ Nevertheless, it has been recently reiterated that it is essential to establish an impact on patients' outcomes if we are ultimately to demonstrate the usefulness of simulations for improved diagnostics, surgical planning or device implantation.^50^ Despite colossal advances on the technical side since those first pioneering Fontan simulations, and despite the undeniable potential for simulations to enrich clinical practice with their predictive capabilities, questions still remain to be answered. Will the uptake of simulation improve clinical success in treating patients with CHD? How cost-effective is the paradigm of embedding computational modelling in the clinical reality? Will the development of patient-specific paediatric devices be supported by the industry? Particularly in CHD, it is possible to learn lessons from computational simulations, and this translational effort is driven at its core by a collaborative effort between engineers and clinicians.
